# Global research trends in radiotherapy for bone metastases: a systematic bibliometric analysis

**DOI:** 10.3389/fonc.2025.1465104

**Published:** 2025-08-29

**Authors:** Fadong Yin, Xuan Liu, Xianxiu Nan, Xianbin Zheng, Xinjue Shi, Jing Yuan, Liu Qiteng, Yuyan Gao

**Affiliations:** Beijing Luhe Hospital, Capital Medical University, Beijing, China

**Keywords:** radiotherapy, bone metastases, bibliometric analysis, CiteSpace, VOSviewer

## Abstract

**Background:**

With the development of various advanced radiotherapy techniques, research related to radiotherapy for bone metastases has made great progress, and scholars have published a large number of publications. In this study, we summarized the knowledge structure of radiotherapy for bone metastases and outlined the research hotspots through bibliometric analysis.

**Methods:**

Publications on radiotherapy for bone metastases from 1992 to 2024 were searched in the Web of Science Core Collection (WoSCC) database. Countries, institutions, authors, references, and keywords in the field were visualized using VOSviewer version 1.6.19 and CiteSpace version 6.3.R1.

**Results:**

1303 publications from 71 countries were included in this study. The number of research publications on radiotherapy for bone metastases has been increasing year by year. The United States of America (USA) ranking first in terms of publication count and co-citation frequency. The most prolific institutions and authors were the University of Toronto and Sahgal A, while Chow E was the most co-cited author. The most co-cited paper was published by Lutz S et al. in 2011 in Internation Journal Of Radiation Oncology Physics. “stereotactic body radiotherapy”, “spine metastases”, “spinal cord compression”, “ immunotherapy” and “oligometastases” are the main keywords of the current research topics.

**Conclusions:**

The application of stereotactic body radiotherapy (SBRT) in the treatment of patients with bone metastases, especially oligometastases, has attracted extensive attention from researchers. How to choose reasonable radiotherapy for patients with complicated bone metastases has now become a research hotspot. Radiotherapy combined with immunotherapy may be the future development trend.

## 1 Introduction

Bone is one of the most commonly metastasized sites of cancer. Autopsy studies have shown that bone metastases are present in up to 75% of cases of breast and prostate cancers (1). The typical presentation of bone metastases is severe pain, restriction of motion, and other symptoms that greatly affect the quality of life of patients. Radiotherapy is minimally invasive and safe, able to relieve pain and reduce subsequent skeletal-related events (SREs) caused by bone metastases, it plays an irreplaceable role in the treatment of bone metastases (2).

Chemotherapy and immunotherapy have advanced considerably in recent years, and the long-term survival rate of patients with breast, prostate, and lung cancers has been significantly prolonged, and the prevalence of bone metastases will continue to increase in the future (3, 4). With the development of imaging technologies such as single-photon emission computed tomography (SPECT) and positron emission computerized tomography (PET CT), the detection rate of bone metastases is also increasing (5–7), and more and more early bone metastases patients have been detected. In addition, the development of advanced radiotherapy techniques, including intensity-modulated radiotherapy (IMRT), stereotactic body radiotherapy (SBRT), and proton radiotherapy, allows the radiation therapist to make treatment tailored to precision radiotherapy.

Based on the above aspects, radiotherapy for bone metastases has increased steadily over time. Bibliometric analysis is a method of information visualization used to identify and summarize the hotspots and frontiers within a field (8). Although studies have used bibliometrics to analyze bone metastases’ comprehensive treatment (9–11). However, there still lacks any bibliometric analysis in the field of radiotherapy for bone metastases. Therefore, the present study is focusing on radiotherapy for bone metastases and researching the trends of development and research hotspots through bibliometric analysis.

## 2 Materials and methods

### 2.1 Data collection

**Figure 1:**
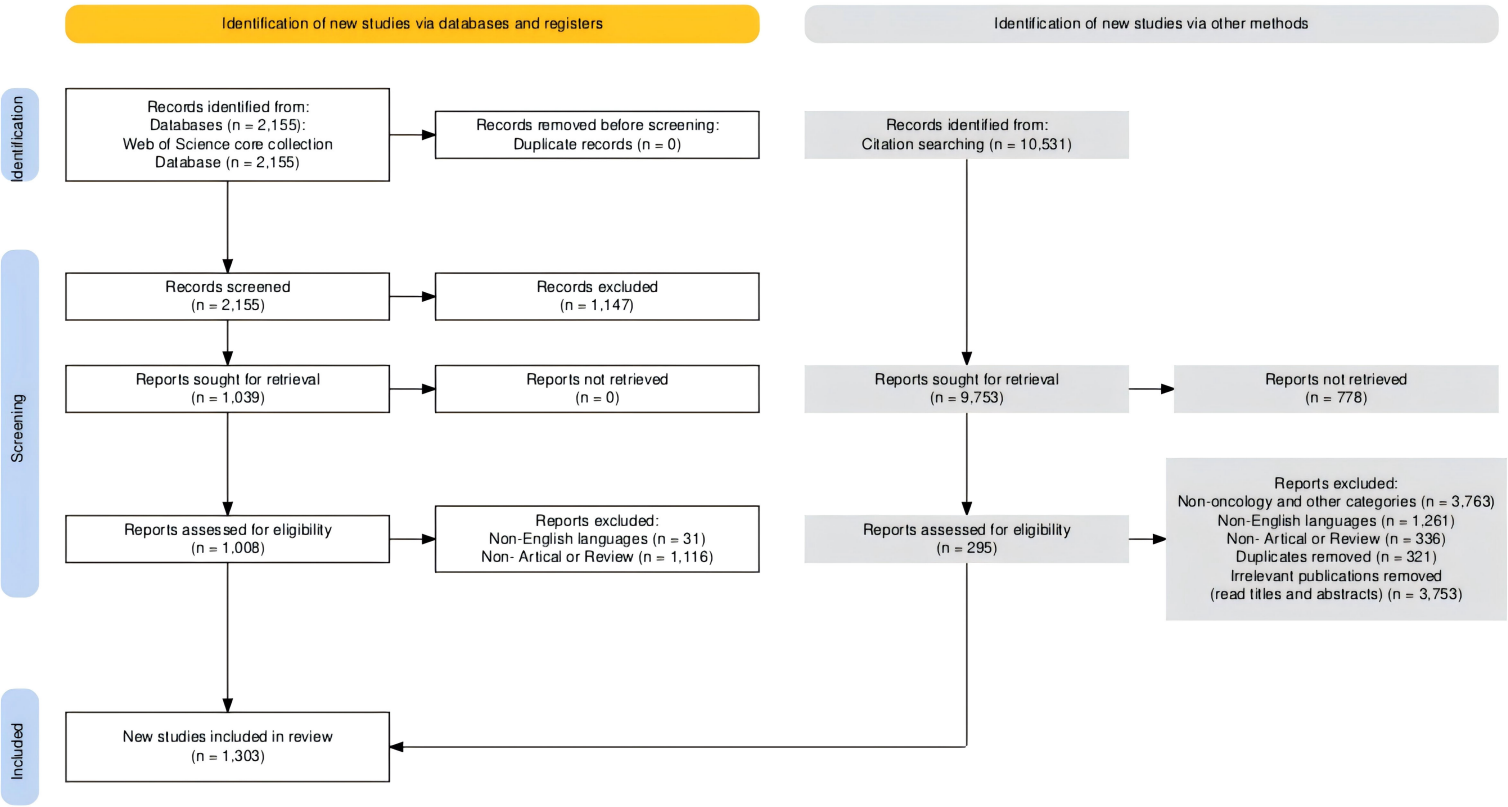
Flow chart of publications selection.

The WoSCC database is the most comprehensive and influential and is often used in bibliometric analysis (11). Therefore, we searched the WoSCC database to find relevant publications on radiotherapy for bone metastases. The search was performed on 27 March,2024 using the retrieval strategy TI="bone metastasis"OR"bone metastases"OR spine OR"spine metastasis"OR"spine metastases"AND radiotherapy OR"radiation therapy"OR radiation OR irradiation OR SBRT OR SABR OR EBRT OR"stereotactic body radiotherapy"OR"stereotactic ablative"OR radiosurgery. Implement the search strategy according to the preferred reporting items for Systematic Reviews and Meta-Analyses (PRISMA) statement (**Figure 1**). In order not to miss any important publication, 10,531 related references were screened to be included. This process was independently reviewed by two authors at the title and abstract level to determine the eligibility of the publication for those publications that were unclear, the full text was reviewed to determine the inclusion.

### 2.2 Bibliometric analysis

Publications obtained by searching using the WoSSC database was exported to plain text format. In this study, VOSviewer version 1.6.19 was used to perform analyses on co-authorship of countries/regions or institutions or authors and keyword co-occurrence analysis (12). Each node indicates an item, the node size indicates the number of publications or the frequency of keyword occurrence, and the color of the node indicates the classification of the items. Meanwhile, the thickness of the line connecting two nodes indicates the strength of the relationship between the two nodes. CiteSpace version 6.3.R1 was used to perform co-citation analysis of journals or authors or references and to generate bibliographic records overlay for journals (13). Microsoft Excel 2019 was used to design basic statistics maps and generate fitted curves for future publication estimates. Geographic distribution maps were generated using Scimago Graphica.

## 3 Results

### 3.1 Global trends of publication outputs

**Figure 2:**
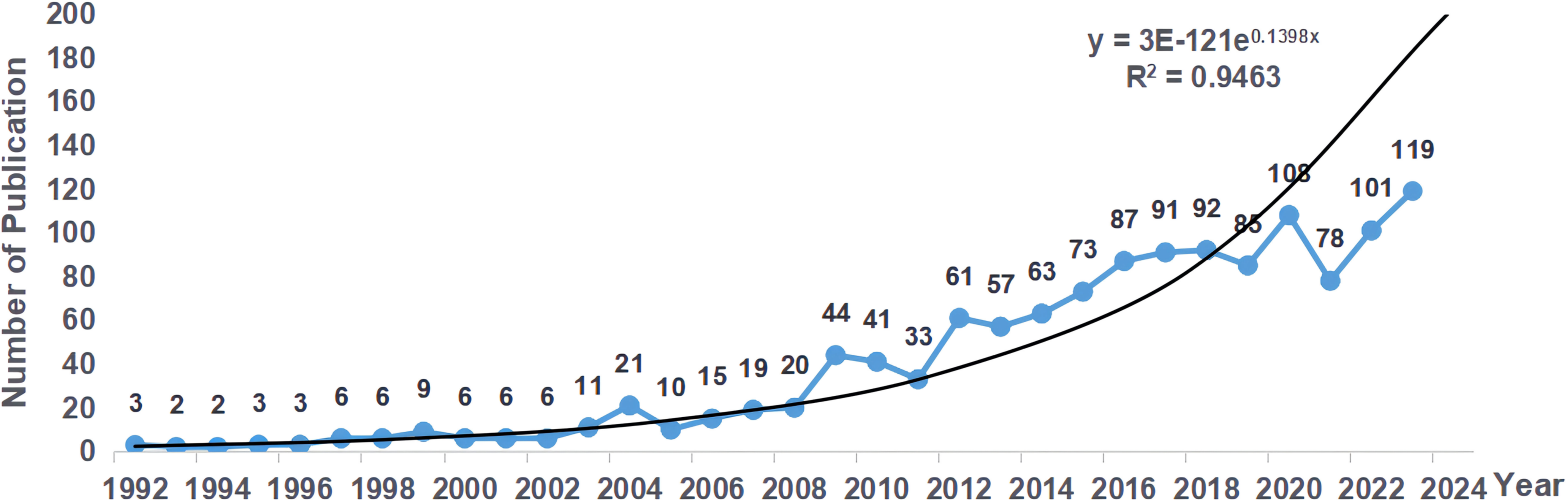
Distribution of annual publications on radiotherapy for bone metastases from 1992 to 2024.

A total of 1303 publications on radiotherapy for bone metastases have been identified, comprising 1152 articles and 151 reviews. Through the publication count, we can see the development trend of the publications about radiotherapy for bone metastases in different periods (**Figure 2**). Generally speaking, the number of publications was relatively small before 2008, and it has grown rapidly since 2009. Although there are small-scale localized fluctuations, the number of annual publications is increasing year by year. In 2023, the number of publications on radiotherapy for bone metastases reached the peak value of 119, and the year of publication has a significant correlation with the number of publications (R²= 0.9463). By the retrieval date, a total of 16 publications were published in 2024. According to the fitted curves, it is expected that more than 200 publications on radiotherapy for bone metastases will be published in 2024.

### 3.2 Countries/regions and institutions

**Figure 3:**
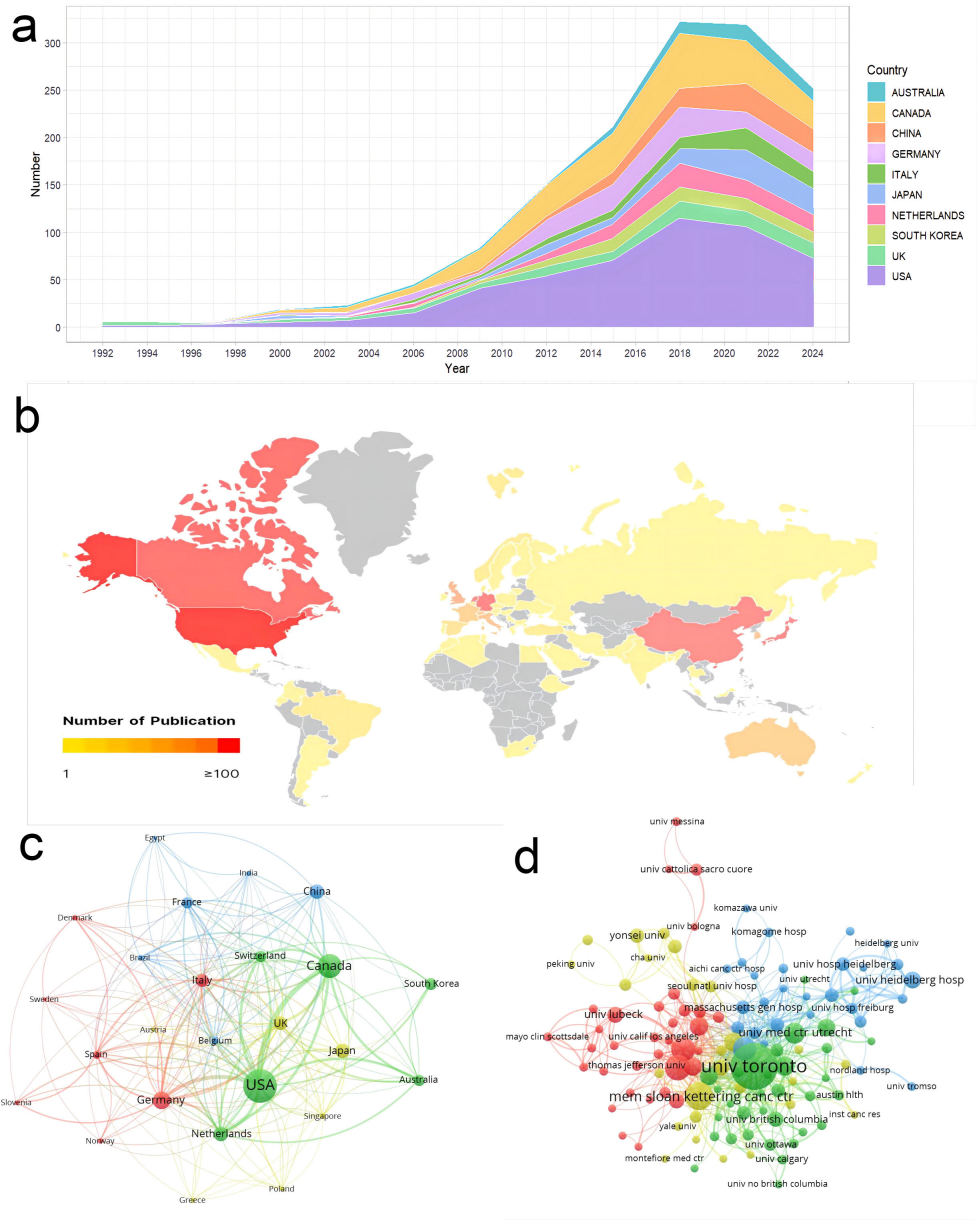
Analysis of publications in countries/regions and institutions. **(a)** Annual publications for the top 10 countries/regions. **(b)** Geographic distribution maps of countries/regions in the number of publications. **(c)** Cooperation network between countries/regions. **(d)** Cooperation network between institutions.

The total publications analyzed in this study came from 71 countries/regions. The USA ranked first with 492 publications, followed by Canada and Germany with 244 and 132 publications, respectively (**Figure 3a**). The top 10 countries/regions are illustrated in **Table 1**. **Figure 3b** shows that publications on radiotherapy for bone metastases are mainly distributed in North America. Canada has the highest average article citation in the world, indicating that the average quality of publications on radiotherapy for bone metastases in Canada is the best in the world.

**Table 1:** The top-10 countries/regions contributed to publications on radiotherapy for bone metastases.

Rank	Country	Documents	Rank	Country	Total citations	Average article citations
1	USA	492	1	USA	12941	34.3
2	Canada	244	2	Canada	8015	42.2
3	Germany	132	3	Germany	2448	21.7
4	Japan	97	4	Netherlands	1482	30.9
5	China	91	5	UK	955	32.9
6	UK	87	6	Japan	938	10.3
7	Netherlands	81	7	Italy	858	15.3
8	Italy	74	8	China	707	8.3
9	South Korea	66	9	South Korea	706	11.8
10	Australia	57	10	France	694	16.1

The thickness of a link on **Figure 3c** reflects the cooperation between countries/regions, and its measurement is called total link strength (TLS). The three countries with the highest TLS are the USA (341), Canada (275), and Germany (174). Besides, the cooperation between Asian countries is weak.

1,817 institutions contributed to publications on the radiotherapy of bone metastases (**Figure 3d**). The top 5 institutions, according to publication count, were the University of Toronto (177), Memorial Sloan Kettering Cancer Center (59), University of Texas MD Anderson Cancer Center (52), Mayo Clinic (48), and University of Pittsburgh (40). Judging by the TLS indicator, the top five are the University of Toronto (427), Queen’s University (135), Mayo Clinic (127), University of Calgary (122), and the University of Texas MD Anderson Cancer Center (109). The University of Toronto takes the leading position in the world for study on the radiotherapy of bone metastases when judged by publication count and intensity of cooperation.

### 3.3 Journals and co-cited journals

**Figure 4:**
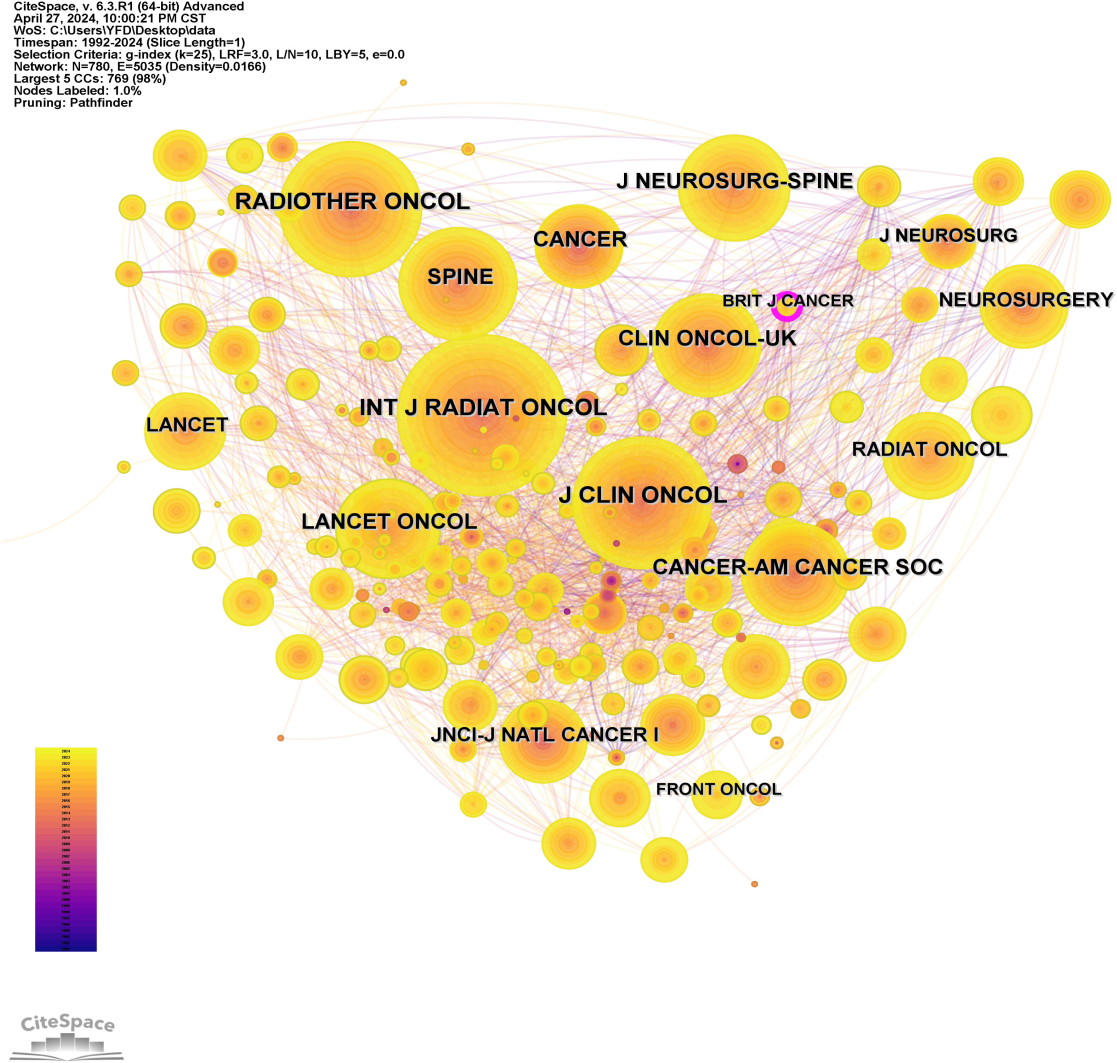
Analysis of journals. The networks of co-cited journals.

Publications on radiotherapy for bone metastases were distributed in 285 journals. The top two journals in the number of publications and co-citations were International Journal Of Radiation Oncology Physics and Radiotherapy And Oncology, both with more than 2000 co-citations. **Table 2** illustrates the top 10 co-cited journals by the most substantial number of publications and co-citations. Journal co-citation is also an important parameter that reflects the status of a journal. We used CiteSpace to generate the journal co-citation network (**Figure 4a**).

**Table 2:** Top 10 journals and co-cited journals on radiotherapy for bone metastases.

Rank	Journal	Documents	Citations	JCR (2024)	IF (2024)	Rank	Co-cited Journal	Citations	JCR (2024)	IF (2024)
1	Int J Radiat Oncol	126	7583	Q1	6.5	1	Int J Radiat Oncol	6163	Q1	6.5
2	Radiother Oncol	73	3254	Q1	5.3	2	Radiother Oncol	2525	Q1	5.3
3	Radiat Oncol	42	604	Q2	3.2	3	J Clin Oncol	1742	Q1	41.9
4	Clin Oncol-Uk	37	1354	Q1/Q2	3.0	4	J Neurosurg-Spine	1492	Q1/Q2	3.1
5	J Neurosurg-Spine	36	1144	Q1/Q2	3.1	5	Cancer-Am Cancer Soc	1554	Q1	5.1
6	Pract Radiat Oncol	31	537	Q1/Q2	3.5	6	Spine	1300	Q1/Q2	3.5
7	Front Oncol	29	187	Q2	3.3	7	Neurosurgery	674	Q1	3.9
8	J Appl Clin Med Phys	26	250	Q2	2.2	8	Lancet Oncol	604	Q1	35.9
9	Strahlenther Onkol	25	587	Q2/Q3	2.5	9	Clin Oncol-Uk	720	Q1/Q2	3.0
10	Med Dosim	24	336	Q4	1.0	10	J Neurosurg	425	Q1	3.6

The dual-map overlay (**Figure 4b**) represents the journal discipline distribution of radiotherapy for bone metastases based on a global context. More than 10000 journals are cataloged in the WOSSC database. This also explains the knowledge flow across disciplines in radiotherapy for bone metastases research. This also explains the citation relationships between the citing journals and cited journals. The citing journals are on the left, and the cited journals are on the right. The two predominant green paths are easy to notice, which indicates the main citation patterns. The findings clearly show that the Medicine/Medical/Clinical journals primarily receive citations from Health/Nursing/Medicine journals or Molecular/Biology/Genetics journals.

### 3.4 Authors and co-cited authors

**Figure 5:**
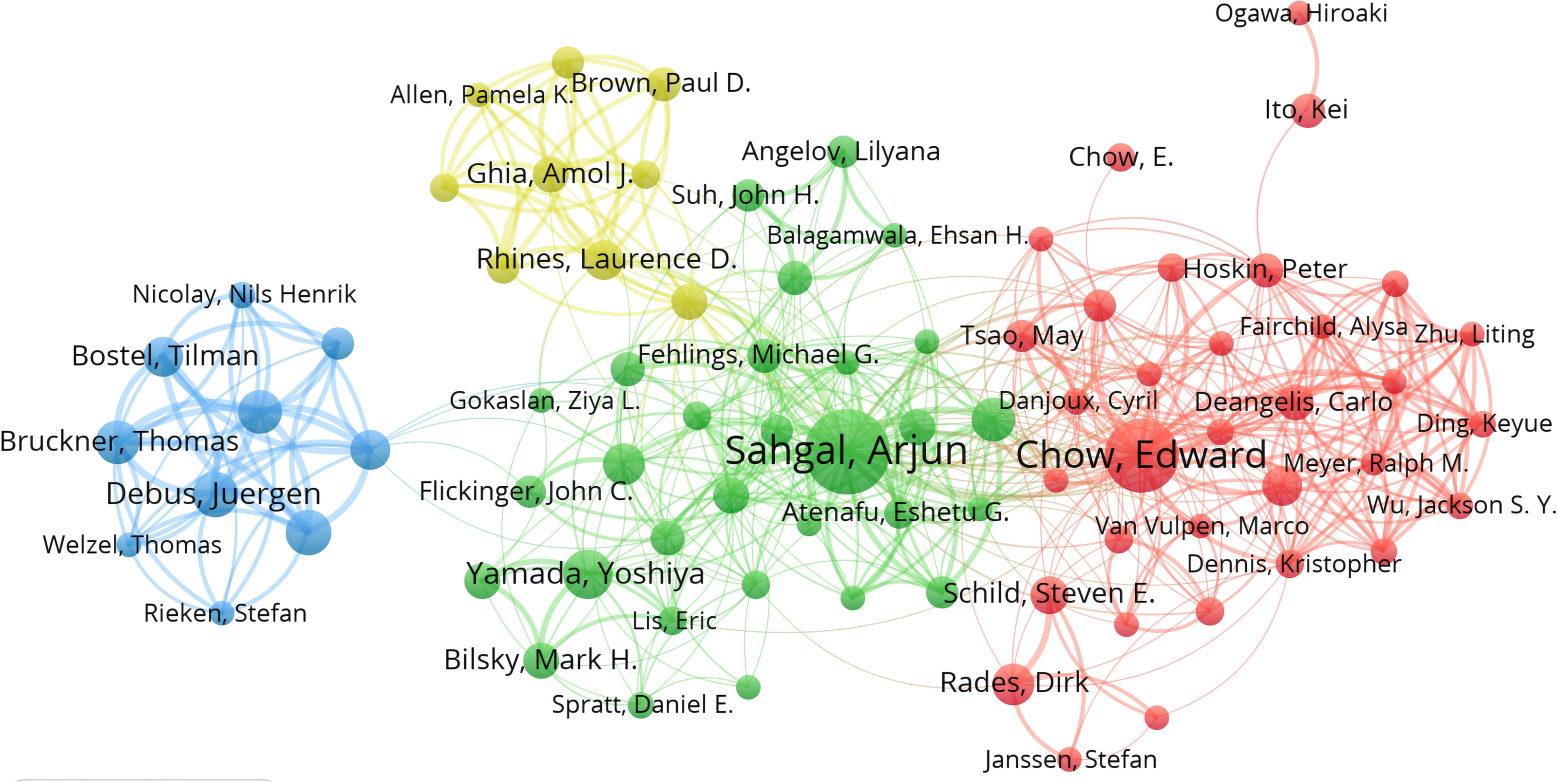
Analysis of authors. Cooperation network between authors.

A total of 6,638 authors contributed publications relevant to radiotherapy for bone metastases. Co-cited authors are two or more authors who are cited together in one or more papers. **Table 3** illustrates the top 10 authors and co-cited authors. Of them, 81 authors published over 10 publications, We used VOSviewer to generate cooperation network between authors (**Figure 5**). Sahgal A was the most prolific author, having published 94 publications, followed by Chow E with 74. Although having published just 27 publications, Gerszte PC was ranked 2nd in co-cited authors (285), with 1,811 total citations.

**Table 3:** The top-10 authors and co-cited authors on radiotherapy for bone metastases.

Rank	Author	Documents	Total citations	Rank	Co-cited Author	Co-citations
1	Sahgal A	94	4667	1	Chow E	484
2	Chow E	74	3857	2	Sahgal A	375
3	Yamada Y	35	2028	3	Gerszten PC	285
4	Debus J	31	636	4	Ryu S	263
5	Rief H	31	650	5	Lutz S	248
6	Soliman H	29	666	6	Rades D	188
7	Schlampp I	28	590	7	Cow BW	186
8	Bruckner T	28	623	8	Yamada Y	180
9	Gerszten PC	27	1811	9	Hartsell WF	179
10	Rades D	27	649	10	Wu JSY	166

### 3.5 Analysis of references

#### 3.5.1 References and co-cited references

Of the 1303 publications related to radiotherapy for bone metastases, those whose citation count exceeded 100 were grouped as highly cited. **Table 4** illustrates the top 10 cited references related to radiotherapy for bone metastases.

**Table 4:** The top-10 cited references related to radiotherapy for bone metastases.

Rank	Title	Journal	Author	Year	Citations
1	Palliative radiotherapy for bone metastases: An ASTRO evidence-based guideline	Int J Radiat Oncol	Lutz S	2011	8015
2	Randomized trial of short-versus long-course radiotherapy for palliation of painful bone metastases	Jnci-J Natl Cancer I	Hartsell WF	2005	2448
3	Radiosurgery for spinal metastases - Clinical experience in 500 cases from a single institution	Spine	Gerszten PC	2007	1482
4	Update on the Systematic Review of Palliative Radiotherapy Trials for Bone Metastases	Clin Oncol-Uk	Chow E	2012	955
5	Meta-analysis of dose-fractionation radiotherapy trials for the palliation of painful bone metastases	Int J Radiat Oncol	Wu JSY	2003	938
6	High-dose, single-fraction image-guided intensity-modulated radiotherapy for metastatic spinal lesions	Int J Radiat Oncol	Yamada Y	2008	858
7	Radiation exposure to the spine surgeon during fluoroscopically assisted pedicle screw insertion	Spine	Rampersaud YR	2000	707
8	International Spine Radiosurgery Consortium Consensus Guidelines for Target Volume Definition in Spinal Stereotactic Radiosurgery	Int J Radiat Oncol	Cow BW	2012	706
9	Palliative radiation therapy for bone metastases: Update of an ASTRO Evidence-Based Guideline	Pract Radiat Oncol	Lutz S	2017	694
10	Palliative radiotherapy trials for bone metastases: A systematic review	J Clin Oncol	Chow E	2007	631

**Figure 6:**
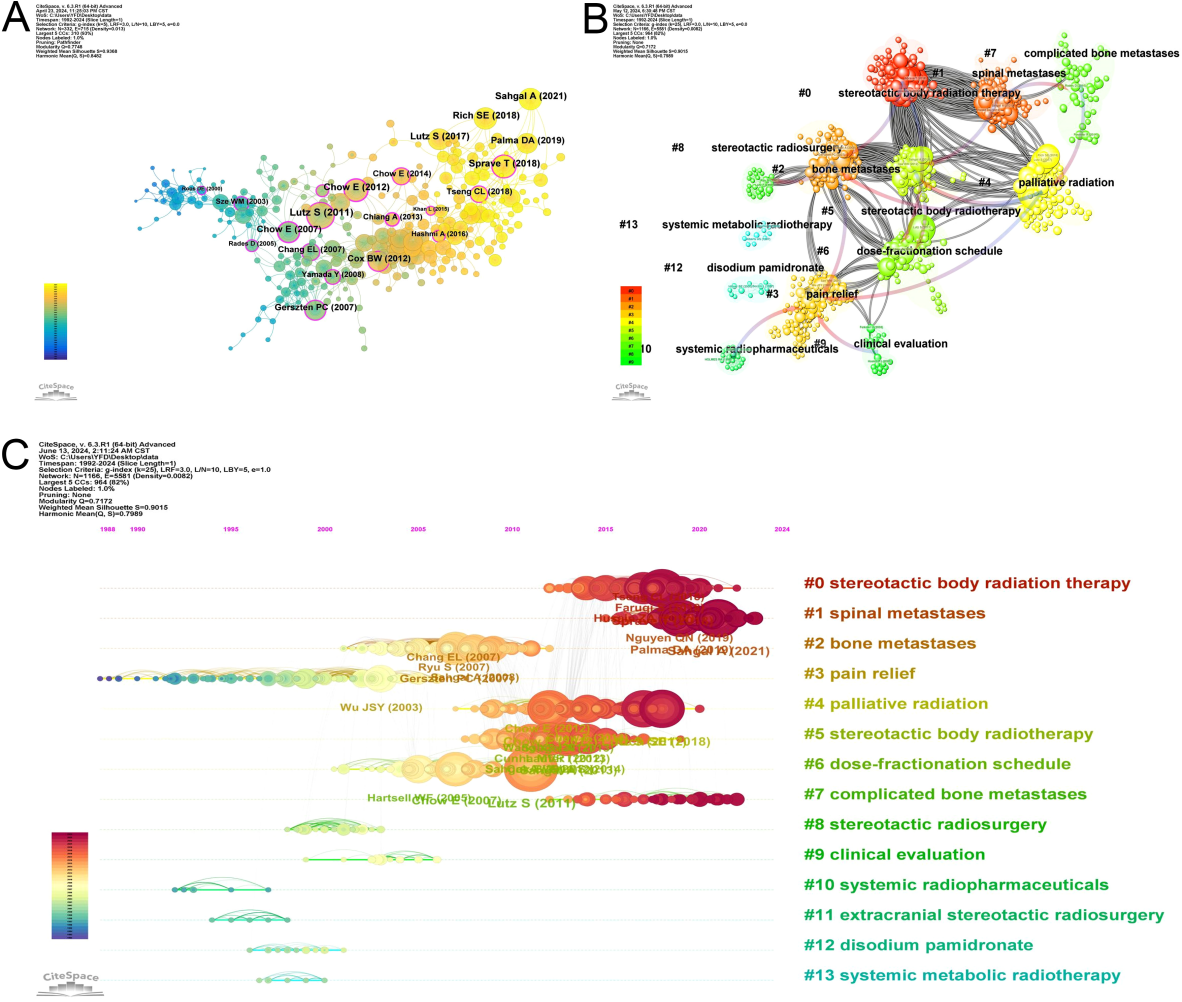
Analysis of references. **(A)** The networks of co-cited references. **(B)** The 14 clusters of the co-cited references. **(C)** Timeline view of the 14 clusters of the co-cited references.

Co-cited reference is a reference that is co-cited in one or more publications (14). This interaction mechanism in the publication grows and contributes to the investigation of the dynamics of development and evolution for a particular discipline. **Figure 6A** illustrates the co-citation network of references whose co-citation exceeding 45.

Co-cited references are typically related to the content and subject. We use the Citespace clustering to find out the research cohesion among these co-cited references. There are 14 clusters presented in **Figure 6B**. The clustering structure is significant as Modularity Q is 0.7172. When Modularity Q exceeds 0.3, clustering is significant, and the distinction among different clusters is more apparent. The Silhouette S of Weighted Mean is 0.9015, indicating high similarity within the same cluster. When this value is more than 0.7, indicates strong clustering with high tightness and similarity within the cluster (14). The blue-to-red arrow is fading, which shows the cross-reference between each cluster. Red arrows identify the knowledge base, and the arrowhead points to the cluster that provides the knowledge. For example, the red arrow connecting #0 (stereotactic body radiotherapy) points to #6 (dose-fractionation schedule) and #8 (stereotactic radiosurgery), which indicates that the knowledge about #0 comes from #6 and #8. **Figure 6C** illustrates that research hotspots in the last few years have focused on #0 (stereotactic body radiotherapy), #1 (spinal metastases), and #7 (complicated bone metastases).

#### 3.5.2 Reference with citation bursts

**Figure 7:**
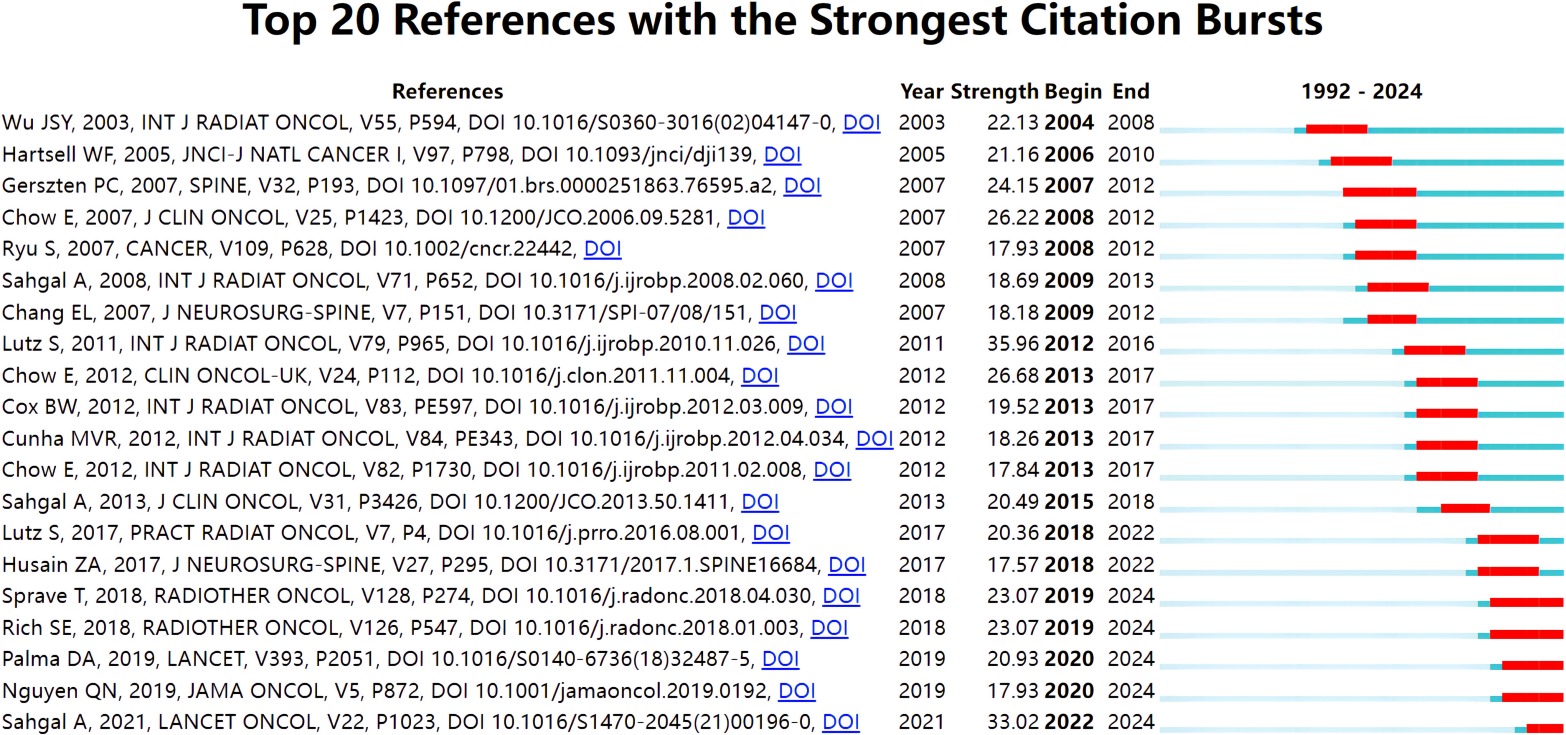
Top 20 reference with the strongest citation bursts.

The top 20 reference with the strongest citation bursts are illustrated in **Figure 7**. The reference with the strongest citation bursts (strength=35.96) was titled “Palliative radiotherapy for bone metastases: an ASTRO evidence-based guideline”, published in *Internation Journal Of Radiation Oncology Physics* by Lutz S et al. (15).This guideline have summarized controversial issues of radiotherapy for patients with bone metastases. The reference with the second strongest citation bursts (strength=33.02) was titled “Stereotactic body radiotherapy versus conventional external beam radiotherapy in patients with painful spinal metastases: an open-label, multicenter, randomized, controlled, phase 2/3 trial”, published in *Lancet Oncology* by Sahgal A et al. (16). This trial reported significant improvement of pain response rates of spinal metastases treated with SBRT as compared to external beam radiotherapy (EBRT).

### 3.6 Analysis of keywords

**Figure 8:**
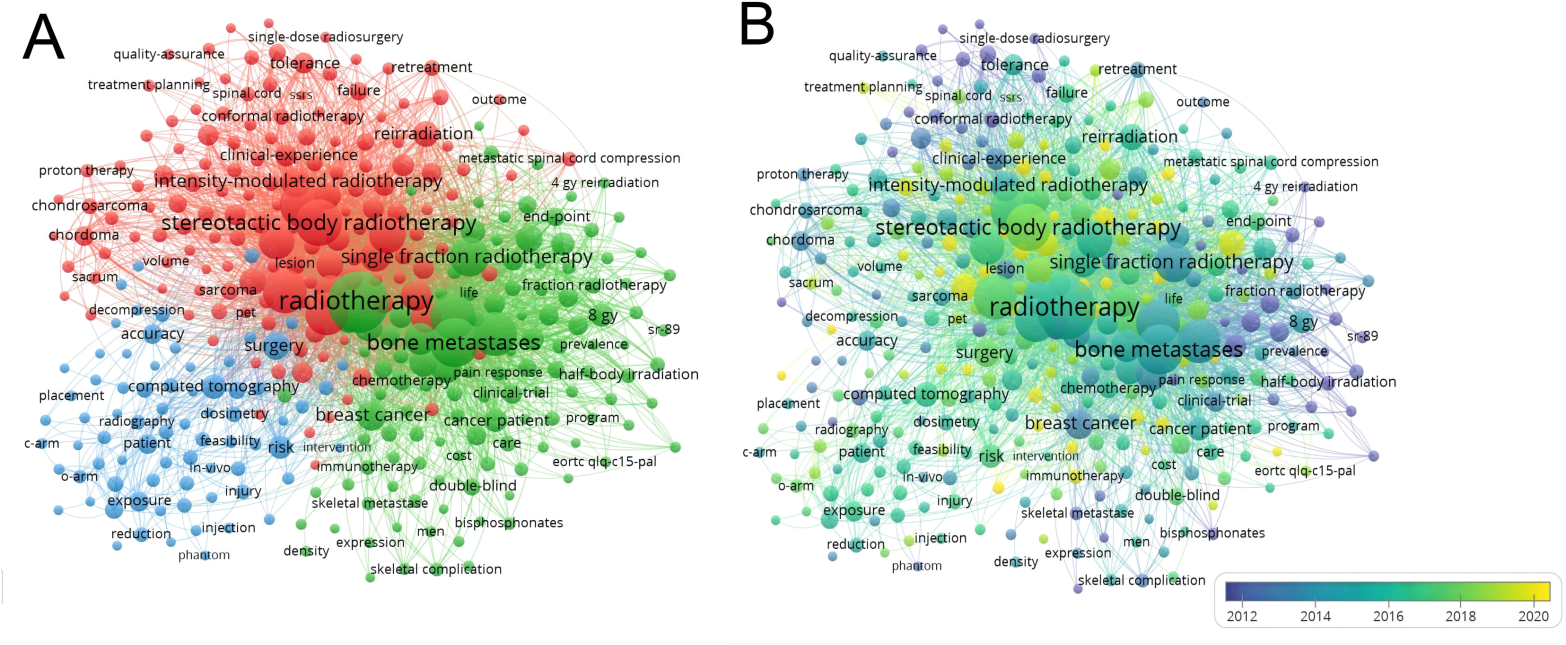
Analysis of keywords. **(A)** Occurrence analysis of keywords. **(B)** The time overlay visualization of keywords.

A total of 3,181 keywords were identified by VOSviewer under the inclusion criteria of at least five occurrences. According to the high-frequency occurrence of 77 synonyms, such as “radiation therapy” “radiotherapy” and “stereotactic body radiotherapy,” the total number of included keywords is 349. The top 20 keywords with high-frequency occurrences are illustrated in **Table 5**. The cluster analysis of the keywords is illustrated in **Figure 8A**, which contains three large clusters. A total of 123 keywords represent the green cluster. Keywords in this cluster include “radiotherapy”, “bone metastases”, “palliative radiotherapy”, “single fraction radiotherapy”, “multifraction radiotherapy” and “external beam radiotherapy” This cluster represents the traditional conventional radiotherapy for bone metastases. A total of 151 keywords represent the red cluster, with keywords such as “stereotactic body radiotherapy”,” radiosurgery”, “intensity-modulated radiotherapy”, “ spine metastases”,” cyberknife” and “oligometastases” This cluster corresponds to the concept of precision radiotherapy for bone metastases. A total of 75 keywords represent the blue cluster and focus on “computed tomography”,” image guidance”,” accuracy”,” exposure” and “diagnosis” This describes imaging diagnosis and evaluation in radiotherapy for bone metastases. The keyword analysis presents breast cancer and prostate cancer as the most researched tumors at present.

**Table 5:** The top-20 keywords on radiotherapy for bone metastases.

Rank	Keyword	Occurrences	TLS*	Rank	Keyword	Occurrences	TLS*
1	radiotherapy	774	5641	11	metastases	175	1369
2	bone metastases	412	3213	12	management	139	1159
3	stereotactic body radiotherapy	369	3007	13	quality of life	125	1038
4	randomized-trial	327	2712	14	pain	120	982
5	radiosurgery	326	2740	15	survival	117	932
6	spine metastases	307	2622	16	prognostic factor	112	914
7	palliative radiotherapy	299	2409	17	disease	109	940
8	cancer	283	2165	18	breast cancer	103	859
9	single fraction radiotherapy	214	1896	19	intensity-modulated radiotherapy	103	849
10	spinal cord compression	181	1631	20	prostate cancer	84	630

*TLS, total link strength, the total co-occurrence frequency between the target keyword and other keywords reflects the importance of related topics in the research field.

The time overlay visualization of keywords (**Figure 8B**) is used to analyze the temporal development of research trends in the field. In this view, colors represent the relative timing of keyword appearance: blue colors represent the first appearances, and yellow colors represent the latest appearances. Finally, the presence of light green indicates recent research hot spots, including “stereotactic body radiotherapy”,” spine metastases”,” spinal cord compression”,” vertebral compression fracture”,” minimally invasive surgery” and “prognostic factors” Now, the trends that were expressed from yellow-colored keywords are the emerging topics, as in “overall survival”,” immunotherapy”, “VMAT”,” instability”, “oligometastases” and “multicenter”.

## 4 Discussion

### 4.1 General information

Through analyzing the number of publications, the trend of developing radiotherapy for bone metastases can be found over 30 years. From a general trend in the years, the first stage was the exploration period before 2003, and the second stage was a full stage of fast development from 2008, especially from 2020 to 2023, with 400 papers published. This number of papers published was significantly improved after the development of the concept of precision radiotherapy.

Among the top 10 countries/regions in terms of the number of publications are all developed countries, except China. And in the top five publication output institutions, only one comes from Canada, others are from the USA. This explains that North America leads the world in the development of radiotherapy for bone metastases. The top five institutions in terms of intensity of collaboration are mainly from the USA and Canada. According to this situation, collaboration is used to motivate the quality and quantity of the publications.

### 4.2 Research trends

Radiotherapy for bone metastases has a rich historical background dating back to the early 20th century, following the discovery of X-rays (17). In the context of reference cluster analysis, “pain relief” emerges as a significant focus. Unlike other tumors, the main goal of treatment for bone metastases is not to reduce the size of the tumor lesion, but rather to provide pain relief to improve the patient’s mobility and quality of life. Early studies lacked consensus on the optimal dose of radiotherapy for pain relief in bone metastases. Subsequently, the dose-response relationship for radiotherapy of bone metastases remained controversial among radiotherapists.

The bibliometric analysis of the high-quality literature on “dose-fractionation schedule” includes Wu et al. (18), Chow et al. (19), Hartsell et al. (20), and Chow et al. (21). All of these studies concluded that there is no significant dose-response relationship for radiotherapy of bone metastases, and there is no significant difference in the overall and complete response rates between single and multifraction fractions. These studies on dose-fractionation schedule have contributed significantly to the knowledge base in the field of radiotherapy for bone metastases.

The central keyword in the red cluster of the keyword analysis is “stereotactic body radiotherapy” and “radiosurgery”. The most highly cited literature on SBRT or radiosurgery (SRS) is a prospective clinical evaluation published by Gerszten PC and colleagues (22). Although the study used the clichéd single radiation treatment, the prescribed dose of the treatment was much greater than 8 Gy (median dose was 19 Gy). Important, this was also the first clinical study that focused on spinal metastases, therefore, represented a landmark study in the field of radiotherapy for bone metastases. Consequently, the research focus would evolve from the different dose-fractionation schedules to the clinical evaluation of SRS or SBRT.

### 4.3 Hotspots and frontiers

The analysis of references and the time overlay visualization of keywords allows quick identification of hotspots and frontiers in radiotherapy for bone metastases. Our results showed that the hotspots are mainly in the following aspects:

#### 4.3.1 SBRT for spine metastases

With the continuous advancement of systemic treatment technologies, the survival rates of cancer patients have significantly improved. To more effectively provide lasting pain relief and prevent further damage to the spinal bones, higher-dose radiotherapy regimens are urgently needed for the treatment of spinal metastases. SBRT is capable of delivering high-dose radiation precisely to spinal lesions while strictly protecting surrounding normal tissues, creating an extremely steep dose gradient at the interface between the spinal cord and the tumor. This not only meets the therapeutic demand for dose optimization in spinal metastases but also ensures the intact function of the spinal cord.

SBRT achieves excellent local control (LC) in the treatment of spinal metastases, particularly in cases of oligometastatic spinal disease. A multicenter retrospective study conducted in Italy reported 1-year and 3-year LC rates of 90.3% and 84.3%, respectively (23). A recent meta-analysis, incorporating six prospective studies on SBRT LC and fourteen on pain response, demonstrated LC rates ranging from 80-95% at 1–2 years in a heterogeneous patient cohort. The overall and complete pain relief rates were 83.2% and 36%, respectively, with a median duration of pain response of 3 months (24). In a randomized controlled trial comparing spinal bone density in patients receiving EBRT versus SBRT, Sprave et al. observed that SBRT increased bone density in lytic metastases, while EBRT showed no significant change (25). However, the incidence of vertebral compression fractures (VCFs) at 3 months was higher in the SBRT group compared to the EBRT group (8.7% vs. 4.3%). The mechanism underlying SBRT-induced VCFs may involve high-dose radiation damaging the bone matrix, subsequently compromising spinal stability following lytic destruction (26). High-dose SBRT can elicit an acute and intense inflammatory response, and given the proximity of metastatic lesions to the spinal cord, inadequate radiation precision markedly increases the risk of myelitis, with potentially more severe consequences compared to EBRT.

The ESTRO clinical practice guideline provides practical recommendations for spinal SBRT based on current clinical evidence (27), including the utilization of specific imaging modalities such as magnetic resonance imaging (MRI) and positron emission tomography (PET/CT). PET/CT and MRI exhibit superior accuracy in identifying macroscopic bone lesions compared to conventional CT imaging. Specifically, in prostate cancer, delineating SBRT target volumes based on 68Ga-PSMA-PET/CT bone uptake can enhance the precision of radiotherapy for prostate cancer bone metastases (28). Recent advancements in software technology have led to the development of dedicated contouring and planning systems that assist clinicians in minimizing the uncertainties associated with the spinal SBRT workflow (29). These technologies maximize the safety and efficacy of spinal SBRT, ensuring optimal patient outcomes.

#### 4.3.2 SBRT versus cEBRT

SBRT has been shown to have the potential to palliate pain caused by bone metastases compared with EBRT (30, 31). However, most of the previous studies have been retrospective or single-arm prospective studies. At present, many scholars are interested in conducting high-quality randomized trials and multicenter collaborative studies to determine whether SBRT could improve pain relief rates compared with EBRT. However, the results of the recent clinical studies of random trials comparing SBRT with EBRT in palliating pain from bone metastases were still indefinite (16, 32–36).

Sahgal et al. (16) reported a better pain relief rate of SBRT than that of EBRT, and in the Discussion section, Sahgal described that the results were different from the RTOG 0631 trial (35). Sahgal attributed this variation to the superior biologically effective doses achieved with multiple-fraction SBRT as opposed to single-fraction SBRT. Conversely, a randomized trial by Pielkenrood et al. (34) found no significant disparities between multifraction SBRT and EBRT. A recent systematic review and meta-analysis comparing the efficacy of SBRT versus EBRT in the treatment of spinal metastases revealed that, while SBRT did not demonstrate superior overall pain response compared to cEBRT, it may be associated with a higher likelihood of achieving complete pain response (37). One study suggested that single-fraction SBRT may offer better LC than multifraction SBRT (38). Consequently, the reasons for the inconsistencies in pain relief between multifraction SBRT and single-fraction SBRT remain unclear. Guckenberger et al. (39) found significantly better pain relief in the multifraction SBRT group. However, this study is different from other series because it used a dose-intensified SBRT of 48.5 Gy in 10 fractions, which is different from the 1–5 fraction commonly used for SBRT in previous reports. It provides a new treatment option for radiotherapy of bone metastases.

Most recent reports only provide the dose prescription, for example, 24 Gy in 2 fractions or 30 Gy in 3 fractions, but often neglect to evaluate coverage and minimum dose. To our knowledge, only Pielkenrood et al. (34) reported that the Clinical Target Volume (CTV) was treated by at least 80% of the prescribed dose, while we are not aware of other studies evaluating the dose received by the tumor. Reports detailed the delivery of 24 Gy in 1 fraction in the context of bone metastases, with 2-year LC of 98% when the D95 of the Planning Target Volume (PTV) was greater than 22.4 Gy. The 2-year LC was only 85% when the D95 was less than 16.5 Gy (40). We speculate that the coverage, too, is related to the rate of remission from pain. Furthermore, it is relevant to consider if the minimum dose is related to the rate of local relapse after pain remission.

Ongoing trials do not provide conclusive evidence that SBRT is superior to EBRT for painful bone metastases, with the caveat that SBRT has been shown to improve overall survival (OS) compared to EBRT, particularly in the oligometastatic setting (41). SBRT also provides more rapid pain relief (32). Oligometastatic bone metastases are likely the best candidates for SBRT (42). In terms of the histology of the metastatic tumor, cancers of the prostate and breast appear to be better responders to radiotherapy for bone metastases than lung cancer (43). Future studies should try to identify the patient characteristics that are associated with the greatest magnitude of benefit so that rational patient selection can be performed in the clinic.

#### 4.3.3 Treatment of complicated bone metastases

Many previous large-scale studies excluded patients with vertebral compression fractures (VCF) or metastatic spinal cord compression (MSCC) (44). A recent report revealed that 34.4% of patients undergoing radiotherapy for bone metastases developed complicated bone metastases (45). Consequently, the issue of complicated bone metastases, particularly those affecting the spine, has garnered significant attention in the academic literature.

The combination of surgery and postoperative radiotherapy remains a viable option for eligible patients. However, there is limited research on the optimal timing of radiotherapy, and no definitive timing has been established (46). Presently, we recommend that 2 weeks between EBRT and surgery be maintained. The option to reduce this interval due to using SBRT without significantly increasing the risk of delayed or impaired wound healing is currently not validated by available data and thus inconclusive on the conclusion (47, 48).

It is suggested that LC in patients with MSCC can benefit from dose-escalating radiotherapy to over 30 Gy in 10 fractions (49). Higher total doses over long treatment durations may offer optimal LC, especially in terms of long follow-up (50). Future research should focus on evaluating the efficacy of high-precision radiotherapy techniques like SBRT and VMAT in managing MSCC. Some studies reported favorable LC with SBRT compared to EBRT (51), but VMAT showed superior therapeutic responses (52, 53).

A lot of research focuses on survival analysis in patients with complicated bone metastases. Such factors that are currently being considered to have an effect on survival in complicated bone metastasis include primary tumor histology, location of bone metastasis, risk of pathological fracture, presence of MSCC, pre-radiotherapy motor status, and expected survival time (54–56). Accurate prediction of the risk of paralysis and prognosis in such patients can help with time intervention and wisely select treatment, thus preventing both overtreatment and undertreatment.

#### 4.3.4 Re-irradiation

The primary objective for most patients with bone metastases seeking radiation therapy is pain relief. However, nearly half of the patients who respond to initial radiation experience pain recurrence within a year (57), and 20% undergo re-irradiation. With the prolonging of survival in patients with bone metastases, active management of pain recurrence or progression becomes increasingly crucial. When considering re-irradiation, patient assessment should encompass three main scenarios: 1) no pain relief or recurrence after initial radiation, 2) patients with partial response to initial radiation seeking enhanced efficacy through further radiotherapy, and 3) radiological disease progression posing a risk of neurological compromise (58).Treatment strategies for re-irradiation may include EBRT and SBRT.

A large systematic review and meta-analysis focusing on non-responsive or recurrent bone metastases reported outcomes for 527 patients re-irradiated with EBRT across seven studies. Re-irradiation was effective in 58% of patients, with a response time of 3–5 weeks and duration of relief lasting 15–22 weeks (59).Although re-irradiation alleviates pain in bone metastasis patients, optimal dose fractionation data are lacking. A randomized controlled trial comparing single-fraction (8 Gy) to multi-fraction (20 Gy) radiotherapy found the single-fraction approach to be more effective with fewer toxicities (60).

For patients previously treated with radiotherapy, the risk of spinal toxicity from re-irradiation is greater than that from initial treatment. Thus, radiation techniques characterized by high conformity and steep dose gradients are advantageous for patients with longer expected survival. Regardless of whether patients initially received conventional radiotherapy (30 Gy in 10 fractions) or SBRT (24 Gy in 2 fractions), SBRT re-irradiation achieved 1-year local control rates of 70-80% without significant spinal toxicity or pathologic fractures (61–63).

While some patients may benefit from re-irradiation, potential toxicity risks must be considered. The extent and timing of disease recurrence, prior radiotherapy regimens, mechanical stability, and overall patient status should be evaluated through multidisciplinary discussion to determine an appropriate re-irradiation plan.

### 4.4 Limitation

This study aims to carry out a comprehensive, systematic review of publications in the area of radiotherapy for bone metastases, carrying out a visualization of software to digest the research trend and grab the hotspot of research of the publications in the WoSSC database. This study, however, has a few limitations. First, this study only selected the WoSSC database for searching, yet at the same time ignored the literature not included in the WoSSC database. Second, the recently published literature, because of its short publication time, has been omitted from this study and has become part of the high-level literature because of its low citation frequency. Different bibliometric analysis software has different calculation methods, which may lead to some bias in the results of the analysis of the same data. Finally, bibliometrics is incapable of reflecting research quality. For example, highly cited studies may have methodological controversies.

## 5 Conclusions

The present study is the first bibliometric study of the publications related to radiotherapy for bone metastases. Our findings revealed a historical view and significant advances in the application of radiotherapy for bone metastases treatment. Currently, breast and prostate cancers are two widely studied malignancies. Current priorities include researching the scope of SBRT’s effectiveness in the treatment of bone metastases, the prognosis of survival in cases of complicated bone metastases, and the synergistic ability of combined radiotherapy with other modalities.

## Data Availability

The raw data supporting the conclusions of this article will be made available by the authors, without undue reservation.
